# Treatment with N- and C-Terminal Peptides of Parathyroid Hormone-Related Protein Partly Compensate the Skeletal Abnormalities in IGF-I Deficient Mice

**DOI:** 10.1371/journal.pone.0087536

**Published:** 2014-02-04

**Authors:** Lourdes Rodríguez-de la Rosa, Ana López-Herradón, Sergio Portal-Núñez, Silvia Murillo-Cuesta, Daniel Lozano, Rafael Cediel, Isabel Varela-Nieto, Pedro Esbrit

**Affiliations:** 1 Instituto de Investigaciones Biomédicas “Alberto Sols”, Centro Superior de Investigaciones Científicas-Universidad Autónoma de Madrid, Madrid, Spain; 2 Unidad 761, Centro de Investigación Biomédica en Red de Enfermedades Raras, Instituto de Salud Carlos III, Madrid, Spain; 3 Instituto de Investigación Hospital Universitario La Paz, Madrid, Spain; 4 Laboratorio de Metabolismo Mineral y Óseo, Instituto de Investigación Sanitaria-Fundación Jiménez Díaz, Madrid, Spain; 5 Red Temática de Investigación Cooperativa en Envejecimiento y Fragilidad, Instituto de Salud Carlos III, Madrid, Spain; 6 Facultad de Veterinaria, Universidad Complutense de Madrid, Madrid, Spain; Inserm U606 and University Paris Diderot, France

## Abstract

Insulin-like growth factor-I (IGF-I) deficiency causes growth delay, and IGF-I has been shown to partially mediate bone anabolism by parathyroid hormone (PTH). PTH-related protein (PTHrP) is abundant in bone, and has osteogenic features by poorly defined mechanisms. We here examined the capacity of PTHrP (1–36) and PTHrP (107–111) (osteostatin) to reverse the skeletal alterations associated with IGF-I deficiency. *Igf1-*null mice and their wild type littermates were treated with each PTHrP peptide (80 µg/Kg/every other day/2 weeks; 2 males and 4 females for each genotype) or saline vehicle (3 males and 3 females for each genotype). We found that treatment with either PTHrP peptide ameliorated trabecular structure in the femur in both genotypes. However, these peptides were ineffective in normalizing the altered cortical structure at this bone site in *Igf1-*null mice. An aberrant gene expression of factors associated with osteoblast differentiation and function, namely *runx2, osteoprotegerin/receptor activator of NF-κB ligand ratio, Wnt3a* **,** *cyclin D1, connexin 43, catalase and Gadd45*, as well as in osteocyte sclerostin, was found in the long bones of *Igf1-*null mice. These mice also displayed a lower amount of trabecular osteoblasts and osteoclasts in the tibial metaphysis than those in wild type mice. These alterations in *Igf1-*null mice were only partially corrected by each PTHrP peptide treatment. The skeletal expression of *Igf2, Igf1* receptor and *Irs2* was increased in *Igf1-*null mice, and this compensatory profile was further improved by treatment with each PTHrP peptide related to ERK1/2 and FoxM1 activation. *In vitro*, PTHrP (1–36) and osteostatin were effective in promoting bone marrow stromal cell mineralization in normal mice but not in IGF-I-deficient mice. Collectively, these findings indicate that PTHrP (1–36) and osteostatin can exert several osteogenic actions even in the absence of IGF-I in the mouse bone.

## Introduction

The insulin-like growth factor (IGF) system, formed by insulin-like peptides, their receptors and binding proteins, plays a central role in the regulation of cell growth and differentiation. Human homozygous loss-of-function *IGF1* gene mutations cause intrauterine and postnatal growth failure and severe sensorineural deafness (OMIM 608747) [1], [2]. Treatment with recombinant human IGF-I has been shown to improve short stature in patients with severe IGF-I deficiency [3], supporting the key role of IGF-I in skeletal development. Moreover, decrease in IGF-I production and/or activity has been suggested to contribute to age-related osteopenia and low bone formation [4], [5]. Mice with a homozygous *Igf1* gene deletion display a 30% size reduction and an aberrant bone phenotype with shortened femoral length and reduction in cortical bone size [6]–[8], and also sensorial impairment [9], as compared to wild type littermates. These bone changes are related to a decrease in both bone formation and bone resorption -with a low number of osteoblasts and osteoclasts-, and also a reduced capacity for osteoblastogenesis and osteoclastogenesis in the bone marrow of *Igf1*-null mice [6]–[8]. Thus, the observation of an increased trabecular bone volume in the proximal tibia of these mice was suggested to be a consequence of the IGF-I effect on osteoclast formation and/or activity at this skeletal site, which is absent in *Igf1*-null mice [6]–[8]. Mice with a homozygous deletion of the gene encoding the IGF-I high affinity receptor (*Igf1r*) show a delayed ossification in the cranial and facial bones, inner ear alterations and die shortly after birth [10], [11]. Furthermore, partial deletion of the *Igf1r* gene causes postnatal growth retardation in humans [12]. IGF1R activation recruits insulin receptor substrates (IRS). Mice with homozygous deletion or spontaneous mutation in *Irs1* show sensory alterations [13]–[15], severe bone growth impairment and low-bone turnover osteopenia [16], [17]. In addition, gain-of-function mouse mutants of IGF-I binding proteins that reduce IGF-bioavailability also consistently show a low cortical and trabecular bone mineral density (BMD) and alterations in bone formation rates [18]–[21].

The bulk of current studies performed in rodents support the notion that the IGF system plays a paramount role in the bone anabolic actions of PTH [22]. Thus, neither *Igf1*-null nor *Igf1r*-null mice show the bone anabolic response triggered by transient administration of PTH in normal mice [23]. IGF1R in mature osteoblasts seems to be a critical PTH target for its skeletal actions [23]. Cells of the osteoblastic lineage are a rich source of PTH-related protein (PTHrP), an important modulator of bone development and bone remodelling [24]. PTH and PTHrP interact with the same PTH type 1 receptor (PTH1R) in osteoblasts [25]. Similarly to PTH, intermittent administration of the N-terminal PTHrP fragment induces bone anabolic features in mice and humans [24]–[30]. Recent findings have also shown that the C-terminal PTH-unrelated region of PTHrP containing the osteostatin epitope (107–111) may also contribute to its osteogenic actions [24], [31]–[35]. Of interest in the frame of the present study, global deletion of *Igf1* [6] or chondrocyte specific deletion of its receptor gene *Igf1r* [36] produces a severe phenotype which resembles that of *PTHrP*-null mice at birth [37], suggesting that PTHrP may signal through IGF-I during bone development. However, the putative contribution of IGF-I to the osteogenic features of the different domains of PTHrP is yet to be explored.

In the present study, we aimed to evaluate whether the response of mouse bone to PTHrP might be affected by the IGF-I status. Specifically, we determined and compared the skeletal effects elicited by PTHrP (1–36) or osteostatin administration to both *Igf1*-null mice and their wild type littermates, at the tissue, cellular, and molecular levels.

## Materials and Methods

### Mouse Genotyping and Functional Characterization

Heterozygous mice with a targeted disruption of the *Igf1* gene were generated and maintained on a hybrid genetic background of MF1 and 129/Sv strains [10]. DNA extraction for genotyping was performed with the REDExtract-N-Amp^TM^Tissue PCR Kit (Sigma-Aldrich, St. Louis, MO), according to the manufacturer’s instructions. PCR primers and conditions used were as previously reported [38], [39]. The animals were fed a standard diet and drinking water ad libitum, and housed following the recommendations of Federation of European Laboratory Animal Science Associations. All animal experimentation was conducted in accord with Spanish and European legislation (EU directive 2010/63/EU) and approved by the Animal Care and Use Committees of Spanish National Research Council (Consejo Superior de Investigaciones Científicas) and Instituto de Investigación Sanitaria-Fundación Jiménez Díaz.

**Figure 1:**
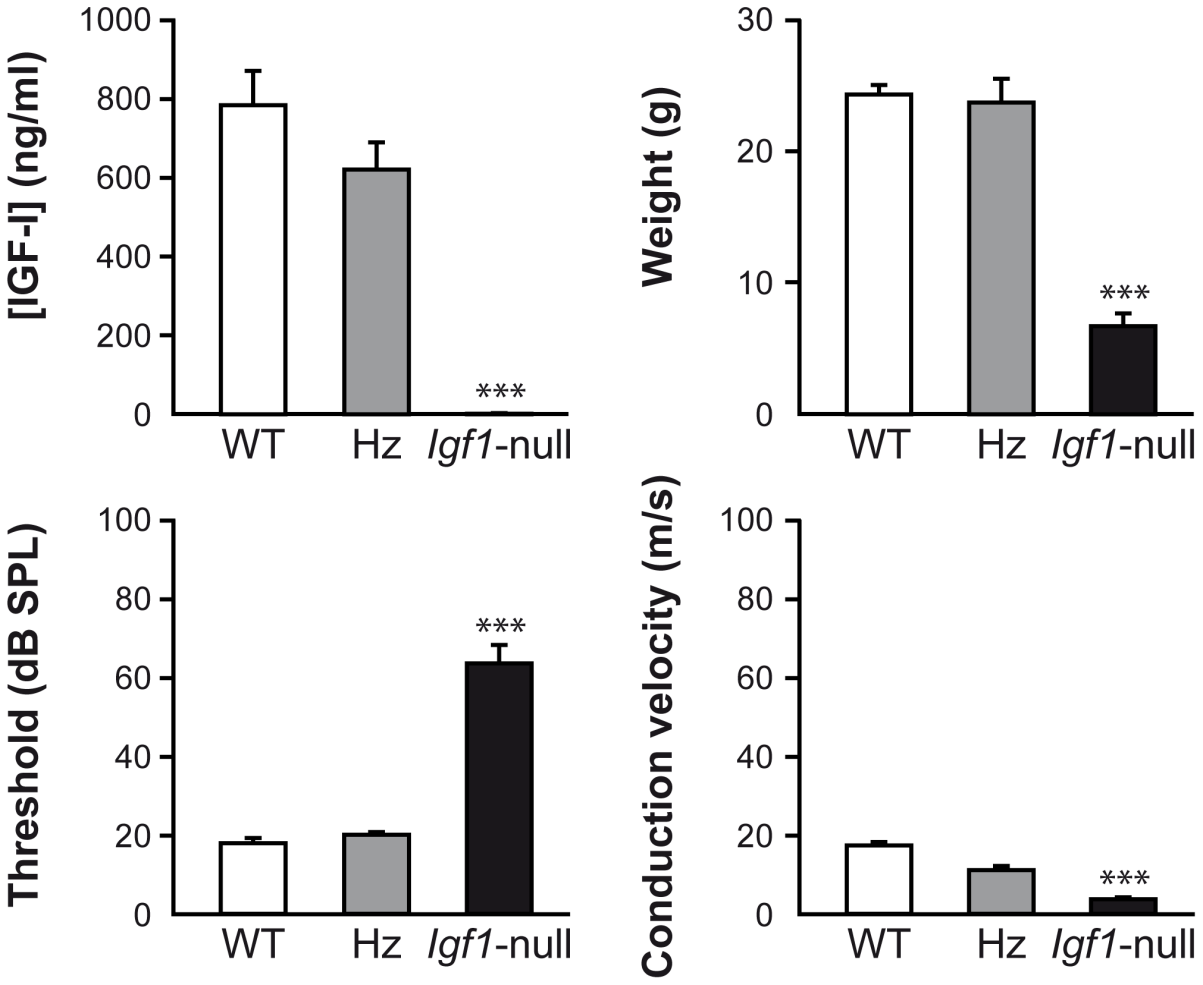
Phenotypic characterization of *Igf1*-null mice. IGF-I serum levels, body weight, as well as auditory brainstem response [hearing threshold, dB sound pressure level (SPL)] and sciatic nerve conduction velocity were measured in young wild type (WT; *Igf1* ^+/+^), heterozygous (Hz; *Igf1* ^+/−^), and *Igf1*-null (*Igf1* ^−/−^) mice, as described in Methods. Data from 2- and 4-month old mice of each genotype were pooled for statistical comparisons. Results are expressed as mean ± SEM corresponding to the following number of mice (males and females, respectively): 7 and 4 (WT); 8 and 4 (Hz) and 2 and 2 (*Igf1* ^−/−^). ***p<0.001 *vs* WT mice.

Initially, young adult (2 and 4 month-old) *Igf1^−/−^* (*Igf1-null*), *Igf1*-heterozygous and *Igf1^+/+^* (wild type) mice were characterized by evaluating serum IGF-I levels, using a standard OCTEIA Rat/Mouse IGF-I kit (IDS Ltd., Boldon, UK), according to the manufacturer’s recommendations [9]. Non invasive tests of neural function, namely auditory brainstem response and sciatic nerve conduction velocity were also performed in these mice, as previously reported [14], [39], [40]. Briefly, auditory brainstem response was evaluated with a TDT System 3™ workstation and the specific software SigGenRP™ and BioSigGenRP™ (Tucker Davis Technologies, Alachua, FL). Determination of sciatic nerve conduction velocity was measured by supramaximal stimulation (9 V, 2 mA, and 0.1 ms) at the sciatic notch and recording at the metatarsian region with a clip electrode. Latencies were measured in each case from the beginning of the stimulus to the first positive wave of the compound muscle action potential. The sciatic motor nerve conduction velocity was calculated dividing the measured latency by the distance from the sciatic notch to the clip electrode. As expected, *Igf1*-null mice showed undetectable IGF-I serum levels, a significantly reduced body weight, severe sensorineural deafness and a reduction in the sciatic nerve conduction velocity when compared to wild type mice. As expected, no differences related to age (within 2 and 4 months) were found in these parameters in each group of mice studied; therefore, pooled data are shown in Figure 1. *Igf1*-heterozygous mice showed a similar functional phenotype to wild type mice and were no further studied. Considering the aforementioned data, we selected two month-old *Igf1*-null and wild type mice for further studies.

### Treatments

Eighteen mice of each *Igf1-null* or wild type genotypes were used. Groups of 6 mice (2 males and 4 females) of each genotype were treated with 80 µg/Kg/every other day of either PTHrP (1–36) or osteostatin subcutaneously for 2 weeks. The control group (3 males and 3 females) of each genotype received the same treatment with phosphate-buffered saline (PBS) vehicle. This time period is within the time frame used for initial characterization of these mice, as described above. Thus, differences observed during 2 weeks (period of study) are unlikely to be accounted for by age but by genotype and type of treatment. Since *Igf1*-null mice have a very poor postnatal survival rate (around 20%) [10], mice from both genders were used *per* genotype and type of treatment. We complied with the 3R (“replace, reduce, and refine”) experimental design recommendation aimed to reduce the number of experimental animals [41]. The number of mice in our study is in the range of those used in related studies [23], [26], [27], [40], [42]. The administered dose of PTHrP (1–36) is similar or lower than that represented by daily PTH administration in other mouse models with IGF-I action deficiency [23], [42], [43], and it has been shown to produce bone anabolism in rodents [26], [28], [44]–[46]. A higher (in molar terms) osteostatin dose was selected, which was similar to that proven to exert anti-resorptive features in mice [47]. Mice tolerated well these treatments, and no secondary effects were observed. Animals were subjected to a 2-week treatment as described above and they were sacrificed 2 h after the last injection of each peptide or vehicle in order to analyze putative rapid changes in gene or protein expression. At the time of euthanasia, both tibiae and femurs were removed, and tissue from individual mice was obtained for histological analysis, total RNA or protein extraction.

### Dual-energy X-ray Absorptiometry (DXA) and μ-computed Tomography (µCT) Analysis

Bone mass changes were assessed in anesthetized mice by DXA (PIXIMUS; GE Lunar Corp., Madison, WI) [28], [48]. Only wild type mice were evaluated since body weight of *Igf1*-null mice (<10 g) lies outside of the PIXIMUS automatic thresholds, as recommended by the manufacturer. Both BMD and BMC were assessed in the same region of interest in each skeletal compartment tested (total body, left femur, and L1–L5 vertebrae) at the start of the study and at the end of treatments using PIXIMUS software. Our animals were young adult and thus growing. Hence, in order to disclose putative bone mass differences between different treatments, BMD and BMC results were presented as percentage of changes as follows: 100×(BMD_14d_-BMD_0d_)/BMD_0d_, and 100× (BMC_14d_-BMC_0d_)/BMC_0d_ (14 d and 0 d subscripts denoting the corresponding values at the end and the beginning of the study, respectively) in each experimental group.

Femurs were scanned using a high-resolution µCT system (Scanco Medical AG, Brüttisellen, Switzerland), with a voxel resolution of 10 µm^3^, an energy of 55 kVp, an intensity of 145 µA and an integration time of 200 ms. Thresholds applied were 392 mg Hydroxyapatite (HA)/cm^3^ and 500 mg HA/cm^3^ for trabecular and cortical bone, respectively. One hundred slices were evaluated in 1-mm of the femoral diaphysis (cortical bone); the number of slices tested in the metaphysis (trabecular bone) varied, given the different bone length in wild type and *Igf1-*null mice, consistently starting at 66% of the femur height down to the growth plate. The three dimensional microarchitectural properties of the cortical bone in the mid-diaphysis and trabecular bone in the distal metaphysis were assessed by using MFEM, R-10 (version V1.2) software (b-Cube AG, Schlieren, Switzerland). The following parameters were calculated: % bone volume/total tissue volume (BV/TV), bone surface (BS), trabecular thickness (Tb.Th), trabecular number (Tb.N), trabecular connectivity density (Conn. D), total area (T.Ar); cortical thickness (Ct.Th) and the polar moment of inertia (J).

### Bone Histology

Mouse femoral samples were fixed in 4% p-formaldehyde in PBS and subsequently decalcified in Osteosoft (Merck, Darmstadt, Germany), dehydrated, and embedded in paraffin. Histological evaluation was carried out by Masson’s staining on sagittal 3 µm sections from each mouse in 4 mice *per* experimental group. The growth plate width in wild type and *Igf1*-null mice was measured with NIH Image J software. Trabecular abundance was also calculated, and expressed as the trabecular number/mm in a bone area below the growth plate. Osteocytes were counted in 4 to 6 random x400-fields per sample in a cortical bone segment between the growth plate and the mid-diaphysis; the corresponding mean score value was normalized to the bone area of each sample. Osteoblasts, identified by their cuboidal shape and localization on bone surfaces and multinuclear osteoclasts (with 3 or more nuclei) [26], [49], were counted in a 0.8-mm^2^ area of the tibial metaphysis immediately below the growth plate. Evaluations were performed by 2–3 independent observers in a blinded fashion for each mouse.

### Bone Marrow Stromal Cell (BMSC) Cultures

To obtain BMSCs, the bone marrow from both tibiae and femurs of either five *Igf1*-null or two wild type mice were pooled and cultured, as previously described [26], [28]. BMSCs were seeded in α-minimum essential medium containing 15% heat-inactivated foetal bovine serum, 1% penicillin–streptomycin, and 2.5 µg/ml fungizone at a density of 1–2.5×10^6^ cells/cm^2^, onto 6-well plates in 5% CO_2_ at 37°C. This culture medium supplemented with 50 µg/ml ascorbic acid and 10 mM β-glycerophosphate (osteogenic medium) was added at day 3 with or without PTHrP (1–36) or osteostatin (each at 100 nM). Half of the volume of the cell-conditioned medium and peptide treatment was exchanged every other day. On day 16, matrix mineralization was determined by staining with 40 mM alizarin red S, pH 4.2, for 10 minutes, and measuring absorbance at 540 nm [28].

### Gene Expression Analysis by Real Time PCR

Femoral samples of each *Igf1*-null and wild type mice were crushed under liquid nitrogen before total RNA extraction with Trizol (Invitrogen, Groningen, NL). For gene expression analysis by real time PCR, cDNA was generated from equal amounts of total RNA obtained from individual mice by reverse transcription (High Capacity cDNA Reverse Transcription Kit; Applied Biosystems, Foster City, CA). Thermal cycling and fluorescence detection was performed on an ABI PRISM 7500 or 7900HT system (Applied Biosystems). TaqMan MGB probes obtained from Assay-by-Design^SM^ or TaqMan® Gene Expression Assays were used (Applied Biosystems) for amplification of: i) osteoblastic genes including runt related transcription factor 2 (*Runx2*), osteocalcin (*OC*), osteoprotegerin (*OPG*), receptor activator of nuclear factor-κB ligand (*RANKL*), and *Pth1r*; ii) canonical Wnt pathway-related genes, *Wnt3a*, cyclin D1 (*Ccnd1*), and *connexin43* (*Cx43*); iii) oxidative stress-related genes, catalase and “growth arrest and DNA-damage-inducible 45 alpha” (*Gadd45*); and iv) genes related to the IGF system and IGF-I signalling targets, including *Igf1*, *Igf*2, *Igf1r*, *Irs2*, tyrosine-protein phosphatase non-receptor type 1 (PTP1B) (*Ptpn1*), and the cell cycle activator forkhead box M1 (*FoxM1*). 18S ribosomal RNA or the mouse ribosomal phosphoprotein P0 (*Rplp0*) was used as endogenous control gene to normalize the expression data obtained. The relative quantification values (RQ) between *Igf1*-null and wild type mice (treated or untreated) were determined by the 2^−ΔΔCt^ method, where ΔΔCt = ΔC_target gene_ – ΔC_reference gene_ [50], and data were expressed as mRNA relative levels *vs* corresponding values in untreated wild type, as reported [38], [49].

### Western Blotting

Mouse intact tibiae from each experimental animal were homogenized in 50 mM Tris-HCl, pH 7.5, 150 mM NaCl, 2 mM EDTA, 2 mM EGTA, 0.2% Triton X-100, 0.3% NP-40, 1 mM dithiothreitol and protease and phosphatase inhibitor cocktail (Sigma-Aldrich) as described [49]. Protein concentration was measured by bicinchoninic acid-based assay (Thermo Fisher Scientific, Rockford, IL) using bovine serum albumin (BSA) as standard. Analysis of protein expression was performed by Western blotting. Equal amounts of protein from individual mice were subjected to sodium dodecyl sulfate–polyacrylamide gel electrophoresis under reducing conditions and transferred to polyvinylidene fluoride or nitrocellulose (for sclerostin) membranes in a Bio-Rad Trans Blot according to the manufacturer’s instructions. After incubation with a blocking solution, the membranes were probed overnight at 4°C with the following primary antibodies: anti-phospho (p)-AKT (Ser473); anti-p-p44/42 extracellular signal-regulated kinase (ERK) 1/2 or anti-p-p38 mitogen activated protein kinase (each at 1∶1000 dilution), and anti-p44/42 ERK1/2 (Cell Signaling Technology; Danvers, MA), or anti-p38α (Santa Cruz Biotechnology; Santa Cruz, CA) (each at 1∶1000 dilution) as loading controls, respectively; and anti-sclerostin antibody (R&D systems; Minneapolis, MN) (0.2 µg/ml). Anti-α-tubulin antibody (Sigma-Aldrich) was used as loading control for the latter primary antibody. Antibodies were diluted in Tris-buffered saline with Tween containing 5% BSA for phosphorylation-specific antibodies or non-fat dried milk for the other antibodies. The membranes were washed and incubated with the corresponding peroxidase-conjugated secondary antibodies for 1 h at room temperature. Immunoreactive bands were visualized by enhanced chemiluminescence (GE Healthcare; Buckinghamshire, UK) using X-ray films, and the bands were quantified by densitometry with NIH Image J software.

### Statistical Analysis

Statistical comparisons of hearing thresholds, NCV and IGF-I serum levels were performed with one factor ANOVA with post hoc Bonferroni or Tamhane test in case of homogeneous or non-homogeneous variances according to Levene’s test, respectively, using SPSS 19.0 (IBM SPSS statistics). Statistical analysis of changes in the expression of IGF system and related genes, assessed by real time PCR, was performed using the Integromics Real Time StatMiner software package (http://www.integromics.com/genomics-data-analysis/pcr-analysis). Statistical significance of IGF system protein expression levels as well as differences in matrix mineralization and bone cell numbers were analyzed by ANOVA with post-hoc Bonferroni test. Other statistical comparisons were done by Kruskal-Wallis test followed by Mann-Whitney test. Results were expressed as mean ± SEM. p<0.05 was considered significant.

## Results

### Changes in Bone Structural Parameters Elicited by PTHrP (1–36) and Osteostatin in *Igf1*-null and Wild Type Mice

**Figure 2:**
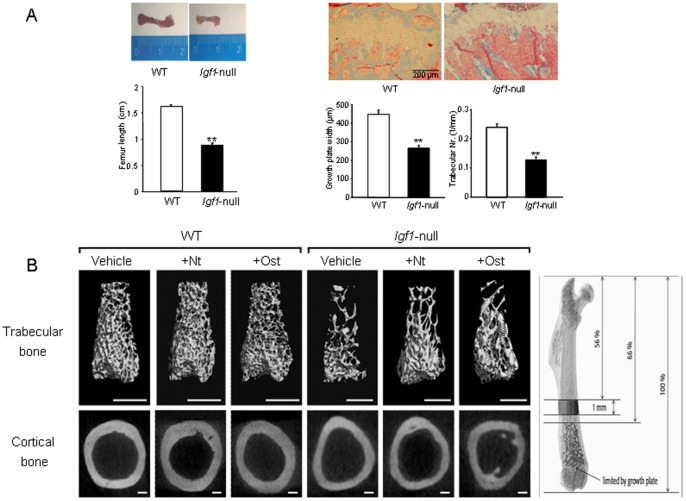
Bone structure in wild type and *Igf1*-null mice with or without PTHrP treatment. (A) Representative images of femur size (left) and the growth plate of the tibia stained with Masson’s trichrome (right) from wild type (WT) and *Igf1*-null mice. Growth plate width, and trabecular number in a defined area below the growth plate, were measured and represented for each genotype. Statistical differences were analyzed by unpaired Student’s t test. Values are expressed as mean ± SEM corresponding to 2 males and 2 females for each genotype. **p<0.01 *vs* WT mice. (B) Representative µCT images from trabecular and cortical regions of the distal femur from WT and *Igf1*-null mice, treated or not with PTHrP (1–36) (Nt) or osteostatin (Ost). Images were adjusted to the same appearance for easy comparison between WT and *Igf1*-null mice which have smaller bones. Thus, scale bars represent 1 and 0.5 mm for trabecular bone (upper panel) or 200 and 100 µm for cortical bone (lower panel) from WT and *Igf1*-null mice, respectively. The regions of interest in trabecular and cortical segments of the mouse bone which were analyzed by µCT are depicted at the top.

*Igf1*-null mice showed a significant decrease in femur length (Figure 2A, left), together with a dramatic decrease in the width of the growth plate and reduced trabecular number in the femoral metaphysis, compared to wild type mice (Figure 2A, right). Moreover, using µCT evaluation, a general alteration was observed in various parameters in both trabecular and cortical compartments in the femur of *Igf1*-null mice compared to wild type mice (Tables 1 and 2 and Figure 2B).

**Table 1:** Changes in trabecular parameters in the distal femur metaphysis from wild type and *Igf1*-null mice, treated or untreated with PTHrP (1–36) or osteostatin.

Trabecular bone	WT	WT+Nt	WT+Ost	*Igf1*-null	*Igf1*-null +Nt	*Igf1*-null +Ost
**BV/TV (%)**	13.48±2.40	10.63±1.01	11.18±0.91	7.04±0.87*	15.70±0.36^#^	10.82±0.54^#,b^
**BS/TV (mm^−1^)**	7.19±1.00	5.64±0.68	6.27±0.54	4.72±0.60*	9.13±0.61^#^	7.37±0.47^#,^ a
**Tb.Th (µm)**	50.37±1.60	56.61±0.71*	54.92±0.85*	44.07±0.70*	52.29±2.23^#^	43.47±1.08^b^
**Tb.N (mm^−1^)**	3.96±0.30	3.12±0.42	3.61±0.38	2.97±0.37*	4.66±0.24^#^	3.68±0.44
**Conn.D (mm^−3^)**	134.31±26.70	96.58±13.66	107.66±13.94	98.72±13.52*	266.08±13.64^#^	172.03±19.21^#,b^

BV/TV, Trabecular bone volume/total tissue volume; BS/TV, trabecular bone surface/total tissue volume; Tb.Th, trabecular thickness; Tb.N, trabecular number; Conn.D, connectivy; Nt, PTHrP (1–36); Ost, osteostatin. Values are mean±SEM corresponding to 2 males and 4 females for PTHrP-treated mice or 3 males and 3 females for vehicle-treated mice of each genotype, respectively**.** Kruskall-Wallis was used to compare differences among all groups, and Mann-Whitney test for comparison between 2 groups.

* p<0.05 *vs* vehicle-treated wild type (WT); ^#^p<0.05 *vs* corresponding vehicle-treated *Igf1*-null;

a p<0.05; ^b^p<0.01 *vs Igf*1-null+Nt.

**Table 2:** Changes in cortical parameters in the distal femur diaphysis from wild type and *Igf1*-null mice, treated or untreated with PTHrP (1–36) or osteostatin.

Cortical bone	WT	WT+Nt	WT+Ost	*Igf1*-null	*Igf1*-null +Nt	*Igf1*-null +Ost
**T.Ar. (mm^2^)**	0.75±0.01	0.87±0.03*	0.87±0.01*	0.27±0.01*	0.31±0.03	0.24±0.01
**Ct.Th. (µm)**	204.5±4.3	223.8±1.4*	222.2±4.0*	130.1±6.5*	126.8±7.7	117.0±2.7
**J (mm^4^)**	0.26±0.01	0.32±0.02*	0.34±0.01*	0.03±0.01*	0.05±0.01	0.03±0.01

T.Ar, total area; Ct.Th, cortical thickness; J, polar moment of inertia. Nt, PTHrP (1–36); Ost, osteostatin. Values are mean±SEM corresponding to 2 males and 4 females for PTHrP-treated mice or 3 males and 3 females for vehicle-treated mice of each genotype, respectively. Kruskall-Wallis was used to compare differences among all groups, and Mann-Whitney test for comparison between 2 groups. *p<0.05 *vs* vehicle-treated wild type (WT).

No significant differences related to PTHrP peptide treatment were observed in body weight in each genotype studied at the end of the study. These values were (g; mean±SEM; n = 6): 26.4±0.5 (wild type, WT); 26.3±1.2 [WT+PTHrP (1–36)]; and 25.9±1.6 (WT+osteostatin); or 8.4±0.4 (*Igf1*-null); 8.4±0.8 [*Igf1*-null+ PTHrP (1–36)]; and 7.8±0.3 (*Igf1*-null+osteostatin). Bone mass differences in wild type mice throughout the study were analyzed by calculating the percentage of change of BMD and BMC values for each mouse, as stated in Materials and Methods. By using this approach, we were able to detect a significant (p<0.05 or lower) increase in these parameters in the femur (but not in the total body or the vertebrae) after treatment with each PTHrP peptide tested. These % values were (mean ± SEM corresponding to 3 males and 3 females for untreated mice, and 2 males and 4 females for PTHrP-treated mice): 0.1±1.2 (wild type, WT); 5.9±2.2 [WT+PTHrP (1–36)]; and 8.1±1.4 (WT+osteostatin) (ΔBMD, g/cm^2^); or 0.1±2.5, 9.2±3.1, 11.8±2.7 (ΔBMC, g), respectively.

By using µCT analysis of the distal femur, differential changes elicited by each PTHrP peptide tested were observed in each IGF-I scenario. Thus, in wild type mice, both PTHrP (1–36) and osteostatin were similarly effective in stimulating cortical parameters, namely T.Ar., Ct.Th and J (Table 1), and also Tb.Th (Table 2) at the femoral metaphysis. Meanwhile, administration of PTHrP (1–36) to *Igf1*-null mice improved all the trabecular parameters evaluated even above control values, but osteostatin treatment was significantly less efficient in this bone compartment. Moreover, neither PTHrP peptide tested showed efficacy to normalize the altered cortical parameters determined in the femur of these animals (Tables 1 and 2). These alterations in the distal femur of mice with different *Igf1* genotype and the osteogenic actions of PTHrP (1–36) and osteostatin are depicted by corresponding µCT images (Figure 2B).

### Comparative Effects of PTHrP (1–36) and Osteostatin on the Expression of Bone Related Factors in *Igf1-*null and Wild Type Mice

**Figure 3:**
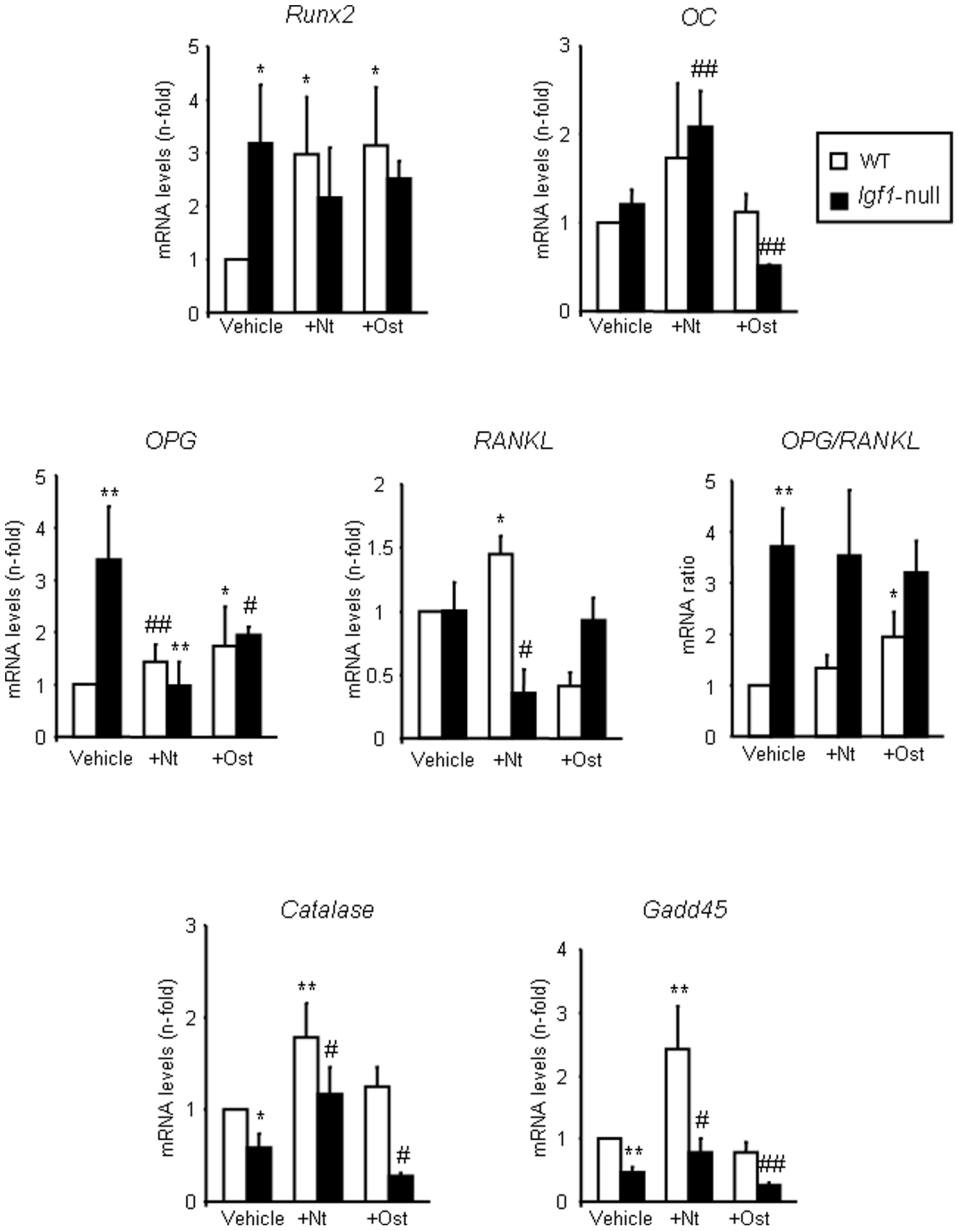
Osteoblast- and oxidative stress-related genes in wild type and *Igf1*-null mice treated or not with PTHrP. Gene expression (assessed by real time PCR) of *Runx2*, *OC*, *OPG*, *RANKL*, *catalase* and *Gadd45* in the femur of wild type (WT) and *Igf1*-null mice, treated or not with PTHrP (1–36) (Nt) or osteostatin (Ost). The variation coefficient of vehicle-WT (control normalized to 1) value was consistently <30% in each case. This variation was taken into consideration for statistical analysis using Kruskall-Wallis test followed by Mann-Whitney test. Values are mean ± SEM corresponding to 2 males and 4 females for PTHrP-treated mice or 3 males and 3 females for vehicle-treated mice of each genotype, respectively. *p<0.05; **p<0.01 *vs* vehicle-treated WT value; ^#^p<0.05; ^##^p<0.01 *vs* vehicle-treated *Igf1*-null mice.

In the femur of wild type mice, treatment with either PTHrP peptide caused a similar increase in the expression of the early osteoblast differentiation transcription factor *Runx2*, but not in that of the late osteoblast differentiation marker *OC* (Figure 3). OPG/RANKL ratio is considered to be a major modulator of bone resorption and bone remodelling [51]. In these mice, only osteostatin treatment enhanced the *OPG/RANKL* mRNA ratio, by increasing *OPG* and decreasing *RANKL* gene expression (Figure 3), coherent with its anti-resorptive features [47]. On the other hand, treatment with PTHrP (1–36) but not osteostatin increased the gene expression levels of *catalase* and *Gadd45* two oxidative stress-associated genes [52] (Figure 3).

In the femur of *Igf1*-null mice, *Runx2* gene expression levels and the *OPG/RANKL* mRNA ratio were increased three times over the corresponding levels of the wild type mice, and were unchanged by either peptide treatment (Figure 3). This observation is consistent with the reported low status of osteoblastic maturation in *Igf1*-null mice [23]. In these mice, PTHrP (1–36) administration increased *OC* mRNA levels, and reversed the down-regulation of *catalase* and *Gadd*45 (Figure 3). *Pth1r* mRNA expression was unchanged in these mice (data not shown), as reported in another *Igf1-*null mouse mutant [23].

**Figure 4:**
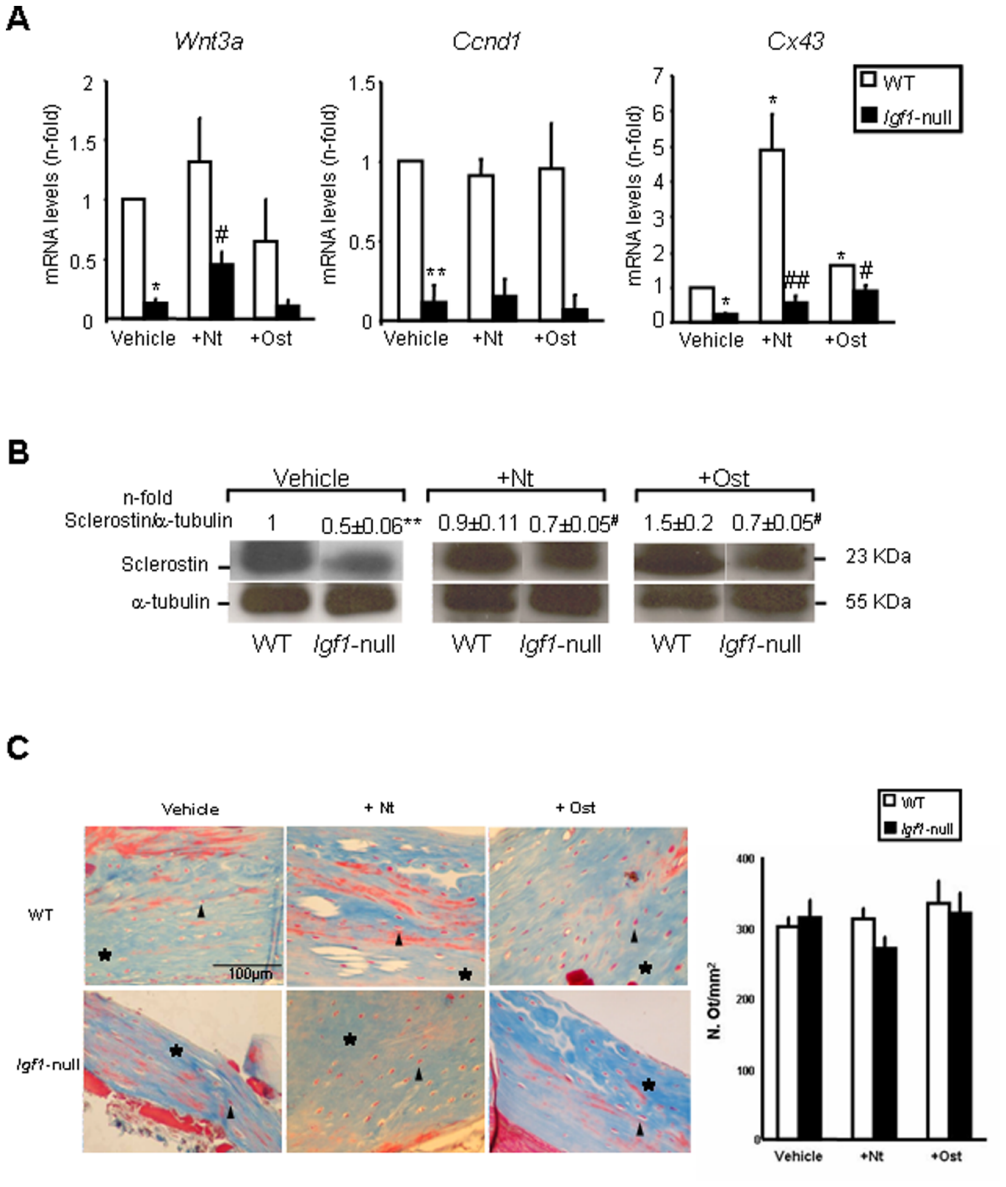
Wnt pathway-related factors in wild type and *Igf1*-null mice with or without PTHrP treatment. (A) Gene expression (assessed by real time PCR) of *Wnt3a*, *cyclin D1* (*Ccnd1*) and *Cx43* in the femur of wild type (WT) and *Igf1*-null mice, treated or not with PTHrP (1–36) (Nt) or osteostatin (Ost). (B) Sclerostin protein levels were assessed by Western blotting in the tibia of the different groups of mice studied. A representative autoradiogram of each experimental condition is shown; values on top represent n-fold ratio over WT value. Values are mean ± SEM, corresponding to 2 males and 4 females for PTHrP-treated mice or 3 males and 3 females for vehicle-treated mice of each genotype, respectively. Vehicle-WT (control normalized to 1) value has a variation coefficient of <20% in each case. Kruskall-Wallis test followed by Mann-Whitney test were used for statistical comparisons. *p<0.05, **p<0.01 *vs* vehicle-treated WT; ^#^p<0.05, ^##^p<0.01 *vs* vehicle-treated *Igf1-*null mice. (C) Osteocyte number (N.Ot/mm^2^) in the cortical tibia in the experimental groups of mice studied. Values are mean ± SEM, corresponding to 2 males and 2 females for each genotype and treatment. Representative images of osteocytes in the cortical mouse tibia (Masson’s staining) from each experimental group of mice are shown. Stars (*) and arrowheads denote bone area and osteocytes, respectively.

Activation of the canonical Wnt pathway is an important mechanism to foster bone formation [53]. We found that the expression of *Wnt3a*, *Ccnd1* and *Cx43* was strongly decreased in *Igf1*-null mice. This decrease was partially compensated for by PTHrP treatment (Figure 4A). Sclerostin is an inhibitor of the canonical Wnt pathway produced by osteocytes, which acts as an important modulator of bone remodelling [54]. We found that sclerostin protein levels were diminished in *Igf1*-null mice; and administration of either PTHrP peptide prevented in part this decrease (Figure 4B). These changes were not related to those in the number of osteocytes in the tibia of *Igf1*-null mice (Figure 4C).

**Figure 5:**
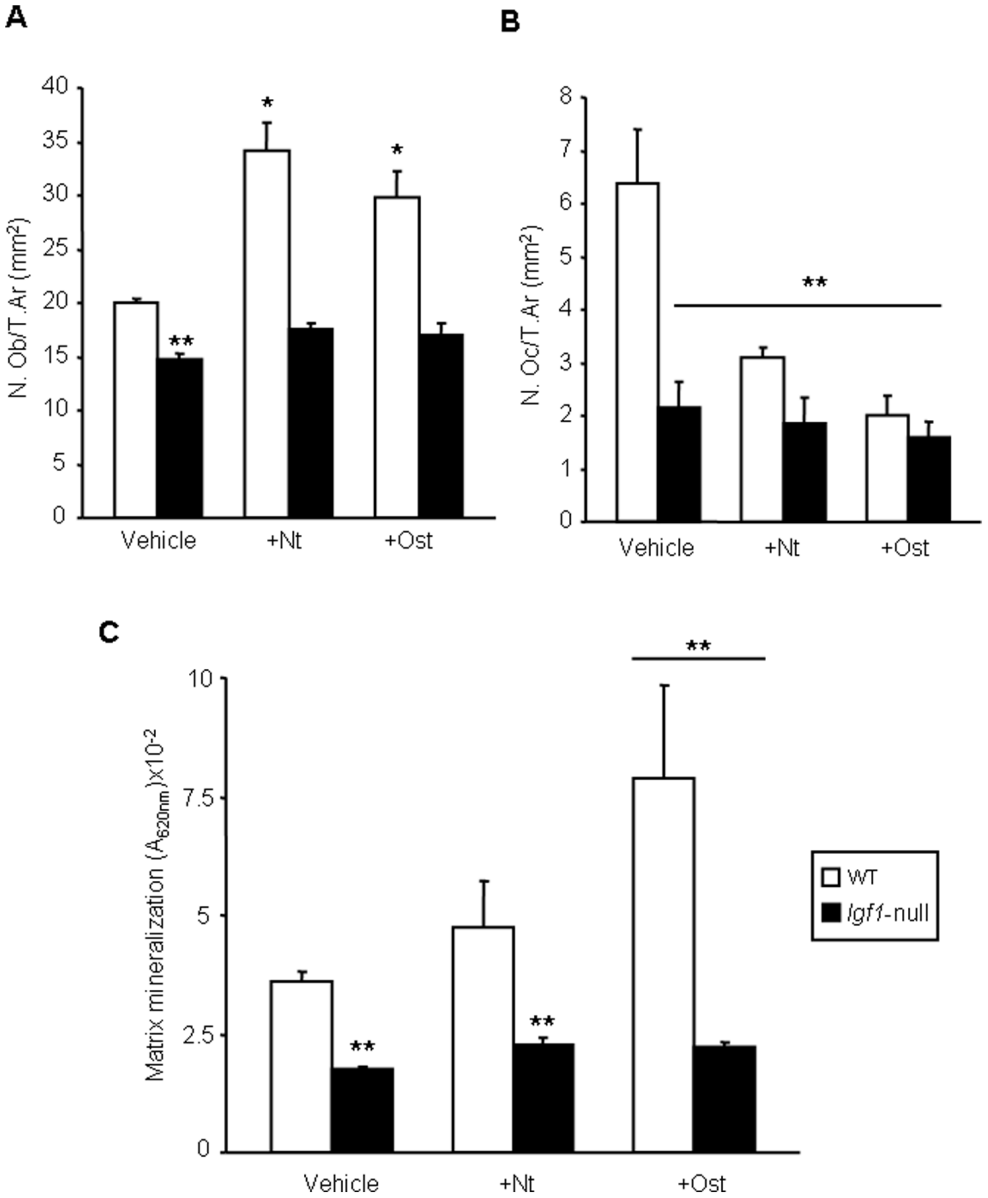
Changes in bone cellularity elicited by PTHrP in wild type and *Igf1*-null mice . Osteoblasts (A) and osteoclasts (B) lining trabecular surface were counted in the tibial metaphysis of mice of both genotypes, treated or untreated with each PTHrP tested, and their number were represented *per* bone tissue area (T.Ar), as described in the text. Values are expressed as mean ± SEM corresponding to 2 males and 2 females for each genotype and treatment. (C) BMSCs from two (1 male and 1 female) wild type (WT) or five (2 males and 3 females) *Igf1*-null mice were cultured for 16 days, with PTHrP (1–36) (Nt) or osteostatin (Ost) (each at 100 nM), or saline vehicle. Matrix mineralization was determined by alizarin staining by measuring absorbance at 620 nm. Values are mean ± SEM (corresponding to 7 culture wells *per* experimental condition). Statistical differences were assessed by ANOVA followed by post-host Bonferroni test. *p<0.05; **p<0.01 *vs* vehicle-treated WT.

We next evaluated whether differences in the observed osteogenic action of each PTHrP peptide according to the mouse genotype were reflected in corresponding changes in osteoblasts and osteoclasts in the mouse tibia. *Igf1*-null mice were found to display a lower amount of both cell types per bone tissue area (mm^2^), compared to wild type mice (Figure 5A and B). Furthermore, the abundance of osteoblasts significantly increased after PTHrP administration in the latter mice but not in *Igf1*-null mice (Figure 5A). On the other hand, either peptide was also effective in decreasing the number of osteoclasts only in wild type mice (Figure 5B).

For assessing whether PTHrP might display cell autonomous actions in an IGF-I deficient environment related to its observed osteogenic actions in *Igf1*-null mice, we used ex vivo BMSC cultures from either these mice or wild type mice. BMSCs from *Igf1*-null mice had a reduced matrix mineralization capacity compared to those from wild type animals. In addition, matrix mineralization significantly increased upon treatment with osteostatin in these cell cultures only from the latter mice (Figure 5C).

### PTHrP (1–36) and Osteostatin Modulate IGF System Expression and IGF Signalling in the Long Bones of *Igf1-*null Mice

**Figure 6:**
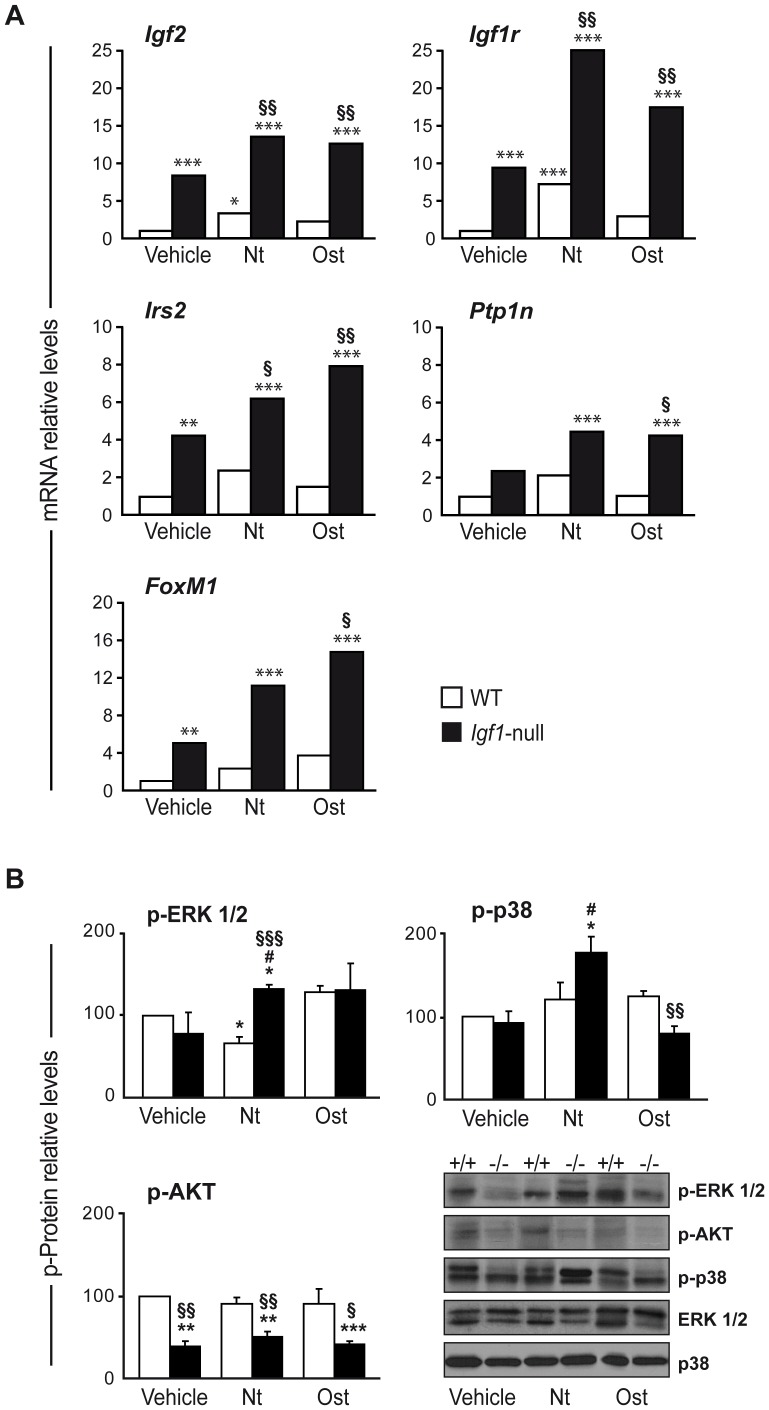
IGF system and signalling targets in bone of wild type and *Igf1*-null mice with or without PTHrP treatment. (A) Gene expression levels of *Igf2*, *Igf1r*, *Irs2, Ptpn1* and *FoxM1* were determined by real time PCR in wild type (WT; white bars) and *Igf1*-null (black bars) mice, treated with either PTHrP (1–36) (Nt), osteostatin (Ost) or saline vehicle. *Rplp0* expression levels were used as endogenous housekeeping control gene. Data correspond to 2 males and 3 females for PTHrP-treated mice or 3 males and 2 females for vehicle-treated mice of each genotype, respectively, and were calculated as log10RQ and represented as relative levels over corresponding WT-vehicle value in each case. Adjusted p-values were calculated with StatMiner software, and were considered significant when p<0.05. (B) Levels of p-ERK1/2, p-AKT and p-p38α were measured by Western blotting in protein extracts from the tibiae of individual wild type and *Igf1*-null mice, treated or not with Nt or Ost. Blots were reprobed for total ERK1/2 or p38α as loading controls. Symbols are: +/+, WT mice; −/−, *Igf1*-null mice. Densitometry values are shown as mean ± SEM corresponding to 2 males and 1 female for PTHrP- or vehicle-treated mice of each genotype, respectively. These values corresponding to vehicle-WT mice were: 0.68±0.07, 0.82±0.05 and 0.62±0.10 for p-ERK1/2, p-AKT and p-p38α, respectively, and were normalized to 100 in each case in the Figure. Statistical significance was estimated by ANOVA and p<0.05 was considered significant. *p<0.05, **p<0.01, ***p<0.001 *vs* vehicle-treated WT value; #p<0.05 *vs* vehicle-treated *Igf1*-null mice; §p<0.05, §§p<0.01, §§§p<0.001 *vs* same treatment in WT mice.

We next evaluated several components of the IGF system and downstream signalling pathways, which might have been targeted by the PTHrP peptides to compensate for the absence of IGF-I. Basal expression of *Igf2*, *Igf1r* and *Irs*2 was found to be increased in the femur of *Igf1*-null mice compared to wild type mice, possibly as a response to IGF-I deficiency. Interestingly, this was not the case for the IGF1R inhibitory tyrosine phosphatase *Ptpn1* gene expression, which remained unchanged. The cell cycle activator *FoxM1* gene was also increased in *Igf1*-null mice. Of note, the expression of these genes further increased in *Igf1*-null mice after treatment with either PTHrP peptide (Figure 6A). Neither PTHrP peptide affected *Igf1* mRNA expression in the femur of wild type littermates (data not shown).

Upon IGF1R activation, mitogen activated kinase-ERK1/2 and phosphatidylinositol-3 kinase/AKT downstream pathways are activated to promote cell proliferation and survival, respectively; whereas the pro-inflammatory p38α kinase pathway becomes inactivated [1], [38]. We here examined the status of these signalling pathways in the tibia of *Igf1*-null mice, and the effect of PTHrP administration to these mice. AKT activation was significantly decreased in the *Igf1*-null mouse tibia, regardless of PTHrP peptide treatment (Figure 6B). On the other hand, there were no significant changes in ERK1/2 and p38α phosphorylation in *Igf1*-null mice compared to wild type mice. Together with *FoxM1* gene expression data, these results suggest that cell proliferation is not severely compromised and could be maintained by the concerted actions of IGF-II and IGF1R in *Igf1*-null mice. Treatment with PTHrP (1–36) increased both p-ERK1/2 and p-p38α levels in the *Igf1*-null mouse tibia. In contrast, in these mice, there was no evident change in the former levels but a reduction in the latter levels upon osteostatin treatment (Figure 6B).

## Discussion

IGF-I-deficient mice have dramatic growth retardation, showing smaller bones than those of normal mice [1], [4]. In this study, young adult mice with *Igf1* gene deletion showed significant alterations in bone mass and bone structure at both cortical and trabecular compartments. Previous studies in IGF-I deficient mice of a different background have shown a decrease in cortical bone formation but an increase of several trabecular parameters in the tibia [6], [8], [23]. It has been hypothesized that bone regional differences in response to IGF-I deficiency might be a consequence of the dual effect of IGF-I on both osteoblastogenesis and osteoclastogenesis [4], [6], [55].

Differences have also been reported for the anabolic action of intermittent PTH or PTHrP (1–36) treatment on trabecular and cortical bone in mice and humans [30], [56], [57]. Here, we show that intermittent PTHrP (1–36) or osteostatin treatment for two weeks in wild type mice showed a similar efficacy at increasing bone mass related to enhancing various cortical parameters but not the majority of trabecular parameters in the femur. In this regard, a previous study has shown that the anabolic effect of PTH was mainly observed in cortical bone, whereas BV/TV was decreased at trabecular level, after 2 week-treatment in male CD1 mice [23]. Moreover, gender differences in PTH bone anabolism have been reported in this mouse strain [58]. Normal male mice on a mixed genetic background of FVB/N, C57Bl/6J and 129Sv also showed a stronger PTH response of cortical bone than trabecular bone after 4 week-treatment [57]. Thus, although anabolic effects of PTH have been reported in both genders of different mouse strains [23], [57], [58], it cannot be ruled out that the osteogenic effect of PTHrP reported here might have been attenuated by combining both genders. A clear anabolic action of PTH in mouse trabecular bone has been reported using a prolonged treatment and/or higher PTH doses than those used here for PTHrP [44], [57], [59]. The observed increase in Tb.Th. by PTHrP administration in the femoral metaphysis of wild type mice could be explained by a possible (yet uncharacterized) effect of these peptides on lining cells as occurs with PTH [60]. Alternately, a putative anti-resorptive action of PTHrP peptides might contribute to this increase, a mechanism supported by our histology data and previous observations [26]–[29], [35], [47]. Further studies are needed to confirm these hypotheses. These findings suggest that differences in mouse strains and/or peptide administration regimes highly influence the bone anabolism achieved by PTH and presumably PTHrP peptides.

Our data indicate an IGF-I dependence for the osteogenic effects of PTHrP peptides on the cortical compartment of the mouse femur. In the present study, the administered doses of PTHrP peptides are higher than those used in a recent clinical study showing the bone anabolism of PTHrP (1–36) in postmenopausal women [30]. We are also aware of the limitation represented by using these high doses for reaching conclusions on the physiological relevance of the present findings. In any event, and consistent with our results, PTH doses in the range of those used here for PTHrP have been reported to be ineffective in the cortical long bones of mice with global IGF-I or osteoblastic IGF1R deficiency [23], [61]. Also of interest, PTH was shown to be ineffective in cortical bone, but not in trabecular bone, in mice with liver-specific IGF-I deletion [62]. The low resorptive activity related to IGF-I deficiency has been suggested to account for the different PTH response of cortical and trabecular bone [6], [23]. In this respect, the reduced number of trabecular osteoclasts in the *Igf1*-null mouse tibia suggests that such a mechanism might facilitate the disclosure of an anabolic action of PTHrP peptides in the trabecular femur of *Igf1*-null mice. Increased OPG mRNA levels (without changes in those of RANKL), which are likely to correlate with those of the corresponding protein [63]–[65], were also detected in these mice. This further supports the notion that a deficit of osteoclastogenesis occurs in *Igf1*-null mice.

Gene expression analysis also suggests the existence of an osteoblast maturation deficit in the absence of a pro-oxidative stress scenario in the femur of osteopenic *Igf1*-null mice. PTHrP (1–36) (but not osteostatin) treatment increased OC gene expression, suggesting it acts toward correcting the altered osteoblast differentiation in these mice. A similar efficacy of PTH in promoting the expression of osteoblast differentiation markers (including OC) in the femur of another *Igf1*-null mouse model has been previously reported [23]. However, the anabolic and catabolic effects of PTH on the proximal tibia of wild type mice were absent in *Igf1*-null mice [23]. This apparent discrepancy was explained by the suggestion that IGF-I might not be essential for proximal events of osteoblast activation (namely, increased expression of some osteoblastic genes) by PTH. In addition, components of the canonical Wnt pathway were found to be affected in the long bones of *Igf1*-null mice, further underpinning the existence of an altered bone formation and remodelling in these animals. The observed decrease in sclerostin protein expression (without changes in osteocyte density) in *Igf1*-null mice indicates a diminished osteocyte function. In this respect, targeted disruption of *Igf1* in mouse osteocytes results in poor Wnt pathway activation causing a marked impairment of bone development and in the bone anabolic response to loading [66], [67]. Our data indicate that administration of PTHrP (1–36) or osteostatin partially recovered Wnt pathway activation in these mice. In fact, PTHrP (1–36) and the osteostatin-related PTHrP (107–139) peptide have recently been reported to target this pathway in osteoblastic cells *in vivo* and *in vitro* [48], [68], related to their osteogenic action in diabetic mice with low bone turnover osteopenia [28], [69].

A previous report has shown that primary calvaria osteoblasts isolated from mice with partial deletion of *Igf1* showed decreased proliferation [70]. In the present study, the long bones of *Igf1*-null mice displayed less trabecular osteoblasts, and BMSCs from these mice had an impaired mineralization capacity. This was similar to previous findings in these cultures from mice with selective deletion of *Igf1r* in mature osteoblasts, which failed to respond to PTH [61], [71]. In line with this observation, BMSCs from *Igf1*-null mice showed a lack of osteogenic differentiation response to PTHrP (1–36) or osteostatin *in vitro*; although this response occurred for osteostatin in BMSCs from wild type mice. However, PTHrP (1–36) was ineffective in this respect in the latter mice, in contrast to a previous report using PTHrP (1–34) and rat BMSCs [72]. Differences in either the N-terminal PTHrP peptide sequence or species (rats and mice) between this study and the present study might explain these discrepancies. Anyhow, our findings support a role for IGF-I in the action of PTHrP on bone marrow osteoprogenitors.

Bone cells synthesize IGF-I and IGF-II [22]; both factors bind the tyrosine kinase receptor IGF1R although with different affinities [1], [73], [74]. Upon this receptor activation, intracellular pathways are activated targeting important cell processes such as proliferation, survival, differentiation, inflammation and stress. Feedback signalling negatively regulates this activation by means of phosphatases and proteases [73]. We here show an increased gene expression levels of *Igf2* and *Igf1r* concomitant to IGF-I deficiency in the mouse femur. Interestingly, an increased expression of docking *Irs*2 was also detected, whilst that of *Ptp1n* was unchanged. In this signalling scenario, ERK activation is maintained whereas that of AKT was decreased, which suggests that cell survival but not cell proliferation would be compromised in the bone tissue of *Igf1*-null mice. These data are in contrast to those obtained in *Igf1*-null cochlea, retina and brain, which do not exhibit an increase in the basal levels of either IGF-II or IGF1R or its substrates [9], [38], [39]. In the cochlea, stress pathways are strongly activated; in contrast, p38α phosphorylation levels were unchanged in the bone tissue of *Igf1*-null mice. Our findings of differential activation of IGF-I signalling mediators between the cochlea and bone of *Igf1*-null mice suggest that IGF-I deficit causes specific alterations in target tissues [4], [74].

Expression levels of *Igf2, Igfr1* and *Irs2* were further increased in the femur of *Igf1*-null mice after treatment with each PTHrP peptide. Although this treatment failed to affect *Igf1* mRNA levels at this skeletal site in wild type littermates (data not shown). In contrast, PTH treatment for a longer period (4 weeks) increased bone IGF-I content in rats [75], [76]. Our data in *Igf1*-null mice also indicate that PTHrP (1–36) was effective in promoting ERK1/2 and p38α phosphorylation, suggesting that these pathways could be effectively modulated through IGF-II/IGF1R. On the contrary, the decreased p-AKT levels in these mice were not normalized after treatment with either PTHrP peptide. Therefore, PI3K/AKT pathway activation by PTHrP in bone seems to be IGF-I-dependent.

These aggregated findings confirm and extend the reported skeletal defects of *Igf1*-null mice using other mouse strains. In addition, our data in mice on a hybrid MF1/129/Sv genetic background support the notion that PTHrP (1–36) and osteostatin can exert osteogenic actions even in the absence of IGF-I.
